# Enhanced Dispersion and Structural Homogeneity in LDPE/Bi_2_O_3_/B_4_C Composites via Cryogenic Ball Milling with Binary-Sized Grinding Media

**DOI:** 10.3390/ma19204262

**Published:** 2026-10-09

**Authors:** Vyacheslav Ivanovich Pavlenko, Roman Nikolaevich Yastrebinsky, Natalia Igorevna Cherkashina, Andrey Ivanovich Gorodov, Dmitry Sergeevich Romanyuk

**Affiliations:** Department of Theoretical and Applied Chemistry, Belgorod State Technological University Named After V.G. Shukhov, 308012 Belgorod, Russiacherkashina.ni@bstu.ru (N.I.C.); gorodov-andreyy@mail.ru (A.I.G.); romanyuk.dmitrij.98@bk.ru (D.S.R.)

**Keywords:** cryogenic ball milling, binary-sized media, LDPE composite, filler dispersion, X-ray phase analysis

## Abstract

The study investigated the effect of a dense two-fraction ball load on the efficiency of cryogenic dispersion and the structural uniformity of polymer composite materials based on low-density polyethylene (LDPE) filled with bismuth oxide (Bi_2_O_3_) and boron carbide (B_4_C). A comparative analysis of two grinding modes—mono-fraction and binary (dense) loading of grinding media—showed that using binary loading reduces the modal diameter of LDPE particles from 19.1 to 13.4 μm, and the number of Bi_2_O_3_ particles smaller than 5 μm increases by 1.25 times. Using XRF and IR spectroscopy, it was established that cryogenic grinding is accompanied by mechanochemical degradation of polyethylene, resulting in the formation of hydroxyl and carbonyl groups, which improve the interfacial interaction between the polymer and inorganic phases of the composite. At the same time, during the subsequent thermopressing of the product, thermal relaxation of the polymer structure occurs and unstable oxidized fragments are eliminated. Energy-dispersive X-ray mapping confirmed a more uniform filler distribution with binary loading: the proportion of element localization regions exceeding 500 μm^2^ decreased for B (0.46 → 0.24), C (0.85 → 0.74), and Bi (0.36 → 0.31). Ultrasonic testing showed a ~3.5% increase in sound velocity (from 2038 to 2110 m/s) and an increase in elastic modulus from 2.36 to 3.34 MPa, indicating improved structural homogeneity and elastic properties. This is the first application of binary-sized grinding media to cryogenic milling of polymer composites. The results demonstrate the potential of cryogenic mechanical activation with a dense two-fraction ball charge for producing highly homogeneous polymer composite materials without organic solvents.

## 1. Introduction

The development of polymer composite materials filled with inorganic dispersed particles is one of the most pressing areas in modern materials science. The popularity of these systems is due to the possibility of obtaining multifunctional composites that combine the lightness and manufacturability of the polymer matrix with the unique physicochemical characteristics of the fillers [1,2]. The introduction of dispersed inorganic phases makes it possible to selectively regulate mechanical strength, wear resistance, and thermal stability, as well as to impart special properties to materials, such as electrical and thermal conductivity, fire resistance, or the ability to shield electromagnetic and ionizing radiation [1,3,4,5]. Furthermore, the development of such compositions opens up great prospects in the field of environmentally friendly technologies and the efficient processing of secondary polymer resources [1,6].

The main obstacle to obtaining composites with specified properties is the difficulty of achieving a uniform distribution of single (non-aggregated) filler particles in the polymer volume. To obtain homogeneous composite systems, mixing methods, mechanochemical and chemical methods (in situ polymerization, sol–gel technologies, coprecipitation), surface modification of fillers and the use of auxiliary substances (surfactants and dispersants) are used [1].

In industrial practice, mixing methods are the most common. Depending on the aggregate state of the components, these may include melt mixing, solution mixing, and solid-state/powder mixing. The melt mixing process requires high temperatures to convert the polymer into a viscous fluid state, which can cause thermal decomposition of both the polymer matrix itself and heat-sensitive fillers. The high surface energy of dispersed particles and their chemical incompatibility with polymers often lead to the formation of aggregates, agglomerates, and clusters instead of uniform distribution. In addition, the high viscosity of the polymer melt hinders the incorporation of nanoparticles and requires significant shear forces to disaggregate them. Thus, the industrial method of melt mixing often does not provide the required degree of uniformity, which negatively affects the performance characteristics of the composite. Mixing in solution, usually carried out at room temperature, ensures a high degree of dispersion of dispersed fillers and makes it possible to overcome the limitations inherent in melt mixing. At the same time, mixing in solution inevitably requires the use of organic solvents, which entails environmental and toxicological problems, an increase in production costs, as well as additional difficulties associated with the handling, regeneration, and disposal of solvents.

The mixing of components in powder form is also accompanied by the formation of aggregates and agglomerates, which subsequently act as stress concentrators and significantly reduce the performance characteristics of the finished product. The problem is exacerbated by pronounced segregation of components, caused by significant differences in the size, shape, density, and surface characteristics of the particles [7,8,9].

A promising direction for solving these problems is the use of mechanochemical methods of processing in the solid phase, for example, high-energy grinding in ball mills or high-speed shear mixing. Cryogenic processing technology is of particular importance, as it transforms the polymer into a brittle state, eliminating its thermal heating and promoting intensive “deformational mixing” of the components at the micron and molecular levels [6,10,11,12]. At cryogenic temperatures (below the glass transition temperature), the polymer behaves as a brittle body, which makes it possible to effectively transfer mechanical energy to break particles and create fresh contact surfaces. The presence of harder and denser inorganic minerals promotes faster polymer dispersion. In this case, the particles of the inorganic additive can act as micro-grinding bodies. They create local zones of high stress, accelerating the breakdown of the soft polymer phase and ensuring intensive mass transfer at the micron level, which leads to an increase in the homogeneity of the system—something that cannot be achieved with conventional mixing. The mechanochemical method makes it possible to combine hydrophobic polymers (for example, polyethylene) with hydrophilic inorganic particles without the use of additional dispersing agents [10].

However, the efficiency of cryogenic ball milling of polymers remains limited due to the configuration of the grinding media. One of the most readily achievable and effective ways to enhance grinding efficiency and control the particle size of the milled product is to adjust the ratio of grinding media [13,14,15]. Research in mineral processing has demonstrated that using a dual-size ball charge (two fractions of grinding media made of the same material but with different diameters) yields a significant increase in grinding productivity compared to a single-size charge. For instance, a simulation study of the ball milling process revealed that binary mixtures of two ball sizes (50.8 mm and 25.4 mm) perform better than mixtures of three sizes (50.8, 35.9, and 25.4 mm), a result attributed to the optimal balance between impact and attrition forces [16]. Study [13] identifies two primary mechanisms of energy transfer in ball mills: the attrition impulse (AI) and the impact compression impulse (ICI). With a maximally dense mono-fraction ball charge (void volume ~26%), the ICI becomes dominant, increasing grinding productivity by 15–20% through more efficient mechanical energy transfer from the grinding media to the material being processed. In stirred mills, the inclusion of a second (smaller) particle size can reduce energy consumption by up to 30% while maintaining grinding efficiency, as the natural segregation of particles of different sizes generates various types of collisions that enhance the grinding of particles of differing sizes [17]. Furthermore, the use of mixtures of grinding media made from different materials (e.g., a mix of steel balls and pebbles or ceramic spheres) has enabled a 13–25% reduction in specific energy consumption compared to grinding in a single-media mode [15,18]. Discrete element modeling has demonstrated that using a mixture of large and small grinding media increases both impact energy and the number of contact points; this results in a more distinct particle size distribution and a finer ultimate particle size compared to using only large or only small media [19]. Specifically, in the grinding of polymetallic cassiterite sulfide ore, a combination of 20 mm and 25 mm steel balls (with a predominance of smaller balls) yielded better results than using media of a single size or a combination with a wider size disparity (20 mm + 30 mm) [20]. This indicates that the ratio of ball diameters is a key parameter for enhancing the technical efficiency of the grinding process.

Although existing research on binary ball mixtures focuses primarily on mineral raw materials [13,14,15,16,17,18,19,20,21,22], this principle can also be applied to polymer systems ground at cryogenic temperatures [23,24]. By adjusting the density of the ball mixtures, one can control the balance between impact and abrasive action—a factor of particular importance for brittle polymers in the glassy state: excessive abrasion leads to unwanted local heating and plastic deformation, whereas a lack of impact impulses reduces the efficiency of fragmentation and surface renewal. However, the available literature contains no references to the use of binary grinding media for the cryogenic grinding of polymers. This study aims to address this gap by systematically comparing a dense two-fraction ball charge with a traditional single-fraction charge during the simultaneous cryogenic milling and mixing of low-pressure polyethylene with Bi_2_O_3_ and B_4_C fillers. The objective is to achieve a high degree of homogeneity and uniform distribution of inorganic particles within the polymer matrix of the composite material through the preliminary mechanical activation of the polymer—specifically, by cryogenically milling the starting powders in a mill utilizing a dense two-fraction ball charge. To achieve this, a binary ball charge was employed for the first time during the cryogenic milling of the polymer composite components. The results revealed a direct correlation between the type of charge and the uniformity of filler distribution at the microscale, thereby confirming the effectiveness of the proposed approach in achieving the stated objective.

## 2. Materials and Methods

### 2.1. Materials

In this study, powdered high-density (low-density) polyethylene (LDPE) of grade 15303-003 (manufacturer LLC Kazanorgsintez, Kazan, Russia) was used as a component of the matrix. The average particle size was 400–450 μm, the polymer purity was more than 99%, the density was 0.920 g/cm^3^, and the melting point was 103–115 °C. Bismuth (III) oxide (Bi_2_O_3_) (supplier—SegmentPro LLC, Moscow, Russia) with a density of 8.90 g/cm^3^, a fraction of 0.1–100.0 μm, and a Mohs hardness of 4.5, as well as boron carbide (B_4_C) manufactured by Platina LLC (Moscow, Russia) with a density of 2.52 g/cm^3^, a particle size in the range of 0.1–120.0 μm, and a Mohs hardness of 9.5, were used as fillers. The choice of fillers was justified by the fact that bismuth oxide (Bi_2_O_3_) and boron carbide (B_4_C) enhance the radiation-protective properties of the composite, which was confirmed in our previous studies [3,25]. Bismuth oxide is used as a shield against gamma radiation, and boron carbide has a high ability to absorb thermal neutrons. The powders were mixed in a ratio of 55 wt.% LDPE, 40 wt.% Bi_2_O_3_, and 5 wt.% B_4_C.

### 2.2. Research Method

When producing composite materials, the preparation of the starting components is a crucial step. The uniformity of mixing the polymer components with denser inorganic particles depends on a number of physicochemical factors and mechanical processing parameters. One significant factor is the uniformity of powder particle sizes, which facilitates a denser (with fewer pores) product formation during subsequent pressing and prevents the aggregation of particles of the same component. For polyethylene, a high degree of dispersion is possible using cryogenic milling.

**Figure 1:**
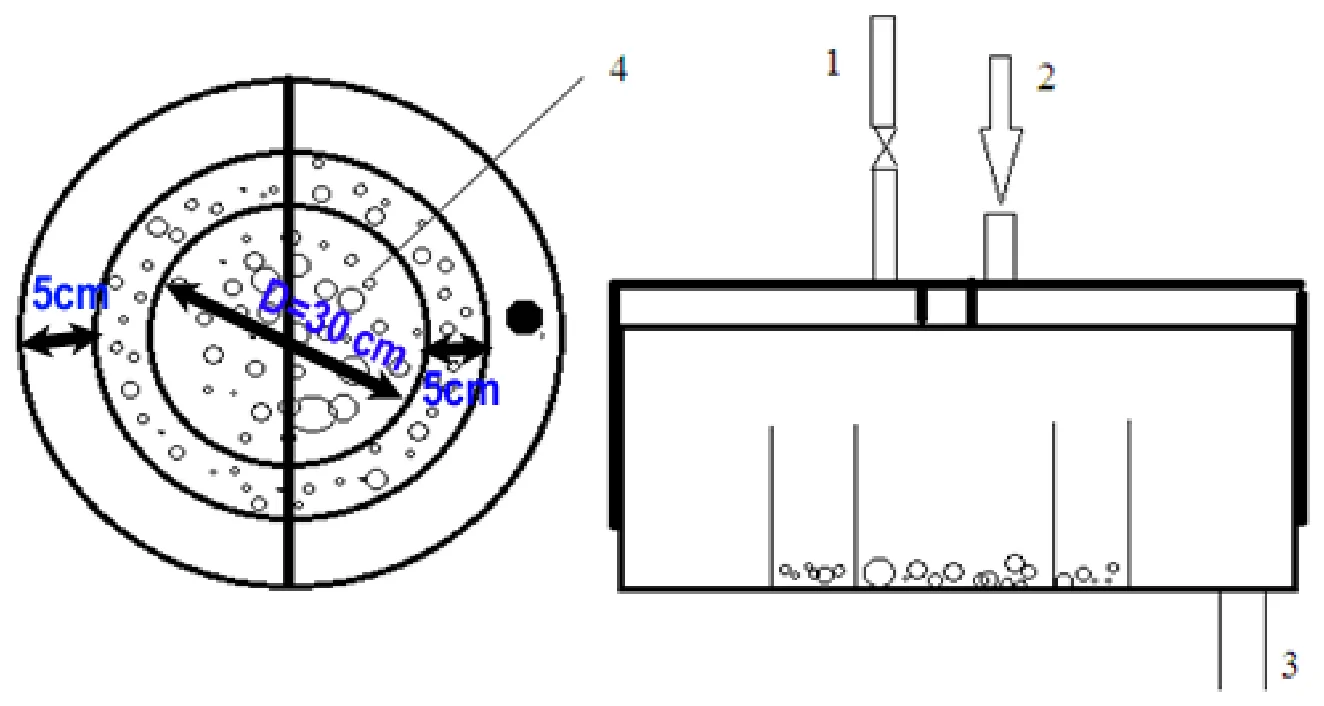
Schematic diagram of a cryogenic mill. 1—Liquid nitrogen feed channel, 2—Material feed channel, 3—Material discharge channel, 4—Grinding media.

The simultaneous grinding and mixing of powdered LDPE with fillers was carried out in a laboratory cryogenic mill consisting of three chambers (Figure 1). The grinding chambers are cylindrical; the first chamber has an internal diameter of 30 cm, while the working zones of the second and third chambers have a thickness of 5 cm each. The mill is 15 cm long. During the grinding process, the material passes sequentially from the first chamber to the second and then to the third through 1 mm diameter openings in the smooth steel partition walls, undergoing changes in particle size distribution and other characteristics. Grinding media—smooth-surfaced carbon steel (St3 grade) balls of various diameters—were loaded into the first and second chambers. The Brinell hardness of the steel balls was 250 kg/mm^2^. To create a dense two-fraction charge (binary-sized ball charge mode), smaller balls were selected to fit into the interstices between the larger balls. The third chamber contained no grinding media but was equipped with special steel blades mounted on the lining—featuring a working surface inclination angle of α = 12°—designed for intensive mixing of the blend following the grinding stage. The grinding process was conducted in batch mode at a rotation speed of 55 rpm.

According to theoretical data [13], there are several types of voids formed by a dense two-fraction load of balls: a tetrahedral void formed by four balls of radius R (the radius of the inscribed ball r = 0.225·R); an octahedral void formed by six balls (r = 0.414·R); a cubic void formed by eight adjacent balls (r = 0.732·R). In this work, balls with a diameter of d = 15 and d = 11 mm (mass ratio of the spheres 2:1) were loaded into the first chamber, intended for coarser grinding, and d = 7 and d = 5 mm (2:1) into the second, which corresponds to the sizes of balls inscribed in the cubic voids. The ratio of the mass of grinding media to the mass of the ground material was 13.75. The mass of the material being ground was 1 kg. The choice of this ratio of the sizes of grinding media and the mass of the ground material was based on the results of preliminary experimental studies.

To create cryogenic conditions (temperatures below −180 °C), liquid nitrogen was fed through the process channel (1). Before comminution, the samples were soaked in the coolant for 15 min to achieve thermal equilibrium throughout the material. The milling process lasted 30 min. Upon completion of the cryogenic cycle, the milled powder was dried to remove condensed moisture by passing an air stream at a temperature of up to 50 °C through the process channel (1).

To produce composites in the form of 50 × 25 × 7 mm plates, the material from the mill was poured into a steel mold and then hot-pressed in a hydraulic press at a specific pressure of 120 MPa and a temperature of 140–160 °C. To compare the obtained results, samples were additionally prepared from powder obtained by milling with balls of the same diameter: in the first chamber, d = 15, and in the second, d = 5 (monosized ball charge mode).

Three independent grinding cycles were performed for each composition. The results are presented as mean values. Differences were considered statistically significant at a significance level of *p* < 0.05. The close agreement of the results from the independent cycles indicates high experimental reproducibility.

### 2.3. Testing Methods

The particle size distribution of the powders was determined by static laser scattering in a dry environment using an Analysette 22 NanoTec plus instrument (Fritsch GmbH, Idar-Oberstein, Germany). Measurements were performed over a particle size range of 0.1 to 2000 μm. Samples were dispersed in a dry air flow using the standard dispersion module. Three semiconductor lasers provided the recording of forward and backscattered radiation: two green lasers with a wavelength of 532 nm and a power of 7 mW for detecting small and ultra-small particles, and one infrared laser with a wavelength of 850 nm and a power of 9 mW for analyzing large particles. For each measurement, 100 consecutive scans were recorded. Scattered radiation was processed using a Fraunhofer optical model.

IR absorption spectra were recorded on a VERTEX 70 Fourier transform spectrometer (Bruker Optics GmbH, Ettlingen, Germany) in the wavenumber range of 4000–400 cm^−1^. The spectral resolution was 0.77 cm^−1^. The interferometer scanning speed (sampling frequency) was set to 5 kHz. Potassium bromide tablets were prepared for analysis. The sample was thoroughly ground with KBr in a 1:100 mass ratio in an agate mortar. The resulting mixture was pressed in a hydraulic press to obtain translucent tablets.

X-ray powder diffraction analysis was performed using X-ray diffraction on an ARL X’TRA diffractometer (Thermo Fisher Scientific SARL, Ecublens, Switzerland). CuKα radiation (λ = 1.5406 Å) was used. The imaging was performed in asymmetric coplanar geometry with a grazing incidence angle of α = 3° (θ scan). Diffraction patterns were recorded over a 2θ range of 4° to 64° with a step of 0.02°. The exposure time at each point was 1 s, and the scanning speed was 16 deg/min. Crystalline phase identification and diffraction maxima indexing were performed using the JCPDF database.

Microscopic examination of the composites was performed using a TESCAN MIRA 3 LMU high-resolution scanning electron microscope (TESCAN ORSAY HOLDING, Brno, Czech Republic) equipped with an X-MAX 50 energy-dispersive spectrometer (EDS) and AZtec software (IE350) (Oxford Instruments NanoAnalysis, High Wycombe, UK) designed for electron probe microanalysis. Prior to analysis, the samples were prepared by cross-sectioning and mechanical grinding. Grinding was performed using SiC sandpaper with various grit sizes (200, 500, 800, and 1000), followed by final polishing with a 1-μm diamond suspension according to the protocol typically used for composite materials. After polishing, the samples were rinsed with distilled water and dried at 50 °C for 3 h. X-ray mapping of sections of polyethylene composite samples with inorganic fillers was performed using a Schottky emission cathode and an In Beam detector in high-vacuum mode (pressure < 10^−3^ Pa) at an accelerating voltage of 5–7 kV. To remove electrostatic charge and prevent drift, a conductive chromium coating ~5 nm thick was applied to the section surface (Cr Kα peaks were excluded from the list of mapped elements in energy-dispersive analysis). Visualization of sections and selection of analysis areas were performed using the contrast of reflected electrons (BSE detector), and final focus adjustment was performed using the secondary electron signal (In Beam detector). The energy-dispersive spectrometer operated in elemental distribution map acquisition mode with an acquisition time ranging from 30 min to several hours (depending on filler concentration) at a fixed dead time of ~30–40%. This ensured reliable separation of the signals from carbon, oxygen, and metal atoms of the composite against the background of the polymer matrix. Image processing of elemental distributions and the determination of the fraction of elemental localization regions with an area exceeding 500 μm^2^ were performed using ImageJ 1.54k software (Wayne Rasband, NIH, Bethesda, MD, USA). To this end, the areas occupied by each element in various regions of the sample were determined automatically. The fraction of elemental localization regions with an area exceeding 500 μm^2^ was calculated as the ratio of the total area of such regions to the total area occupied by the corresponding element.

The porosity of the composite was assessed by flaw detection using a Pulsar-1.2 ultrasonic flaw detector (Moscow, Russia) based on changes in the speed of sound in composite materials. The instrument’s operating principle is based on measuring the transit time of an ultrasonic pulse through the product material from the emitter to the receiver. The ultrasonic speed is calculated by dividing the distance between the emitter and receiver by the measured time. Measurements were conducted for five samples of each composition. Based on the sound speed data in the composite materials, the elastic modulus values of the samples were calculated using the formula

$$E=\frac{\gamma \cdot V^{2}}{\varphi }\times 10^{3}$$

where *E* is the modulus of elasticity (Pa), *V* is the numerical value of the ultrasound speed (m/sec), *γ* is the bulk density (g/cm^3^), and *φ* is the coefficient.

## 3. Results and Discussion

**Figure 2:**
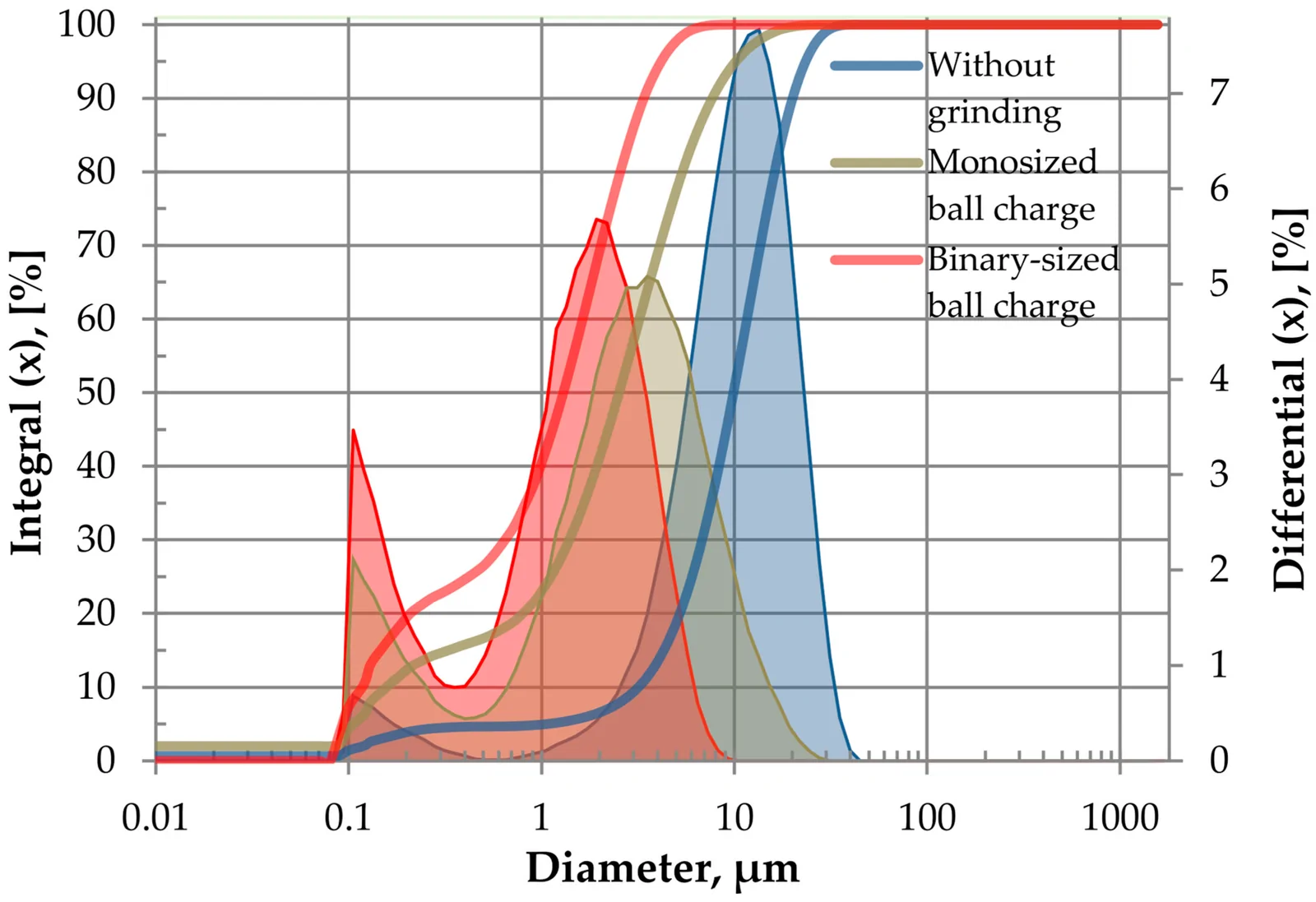
Granulometric composition of the initial Bi_2_O_3_ powders and the products of their grinding in a cryogenic mill with two types of ball loading.

The particle size distribution of the initial Bi_2_O_3_ powder and the products of its milling in a cryogenic mill using a dense two-fraction ball charge (binary-sized ball charge), as well as with a load of balls of a single diameter (monosized ball charge) is shown in Figure 2. Most of the initial bismuth oxide particles (approximately 90%) have sizes from 1 to 20 μm. The main distribution range is at particle sizes of approximately 10 μm, which is evident from the sharp increase in the integral curve in this region. The proportion of the submicron fraction (<1 μm) is insignificant. Particles larger than 20 μm are rare. A small proportion of particles, approximately 50 μm in size, is present. After cryogenic milling of bismuth oxide powder, both in the binary-sized ball charge and monosized ball charge modes, a more pronounced bimodal distribution appears. A significant difference is observed in the position of the fundamental mode: when using a dense charge, it shifts to the 2.3 μm region, whereas when grinding with balls of the same diameter, the fine-dispersed fraction is noticeably less pronounced. The percentage of particles smaller than 5 μm after grinding with a dense charge was 97.9%, which is 1.25 times higher than the corresponding figure for the monosized ball charge mode (77.8%). Thus, the use of a dense, two-fraction grinding media ensures more intensive grinding of bismuth oxide and the formation of a significantly finer product.

**Figure 3:**
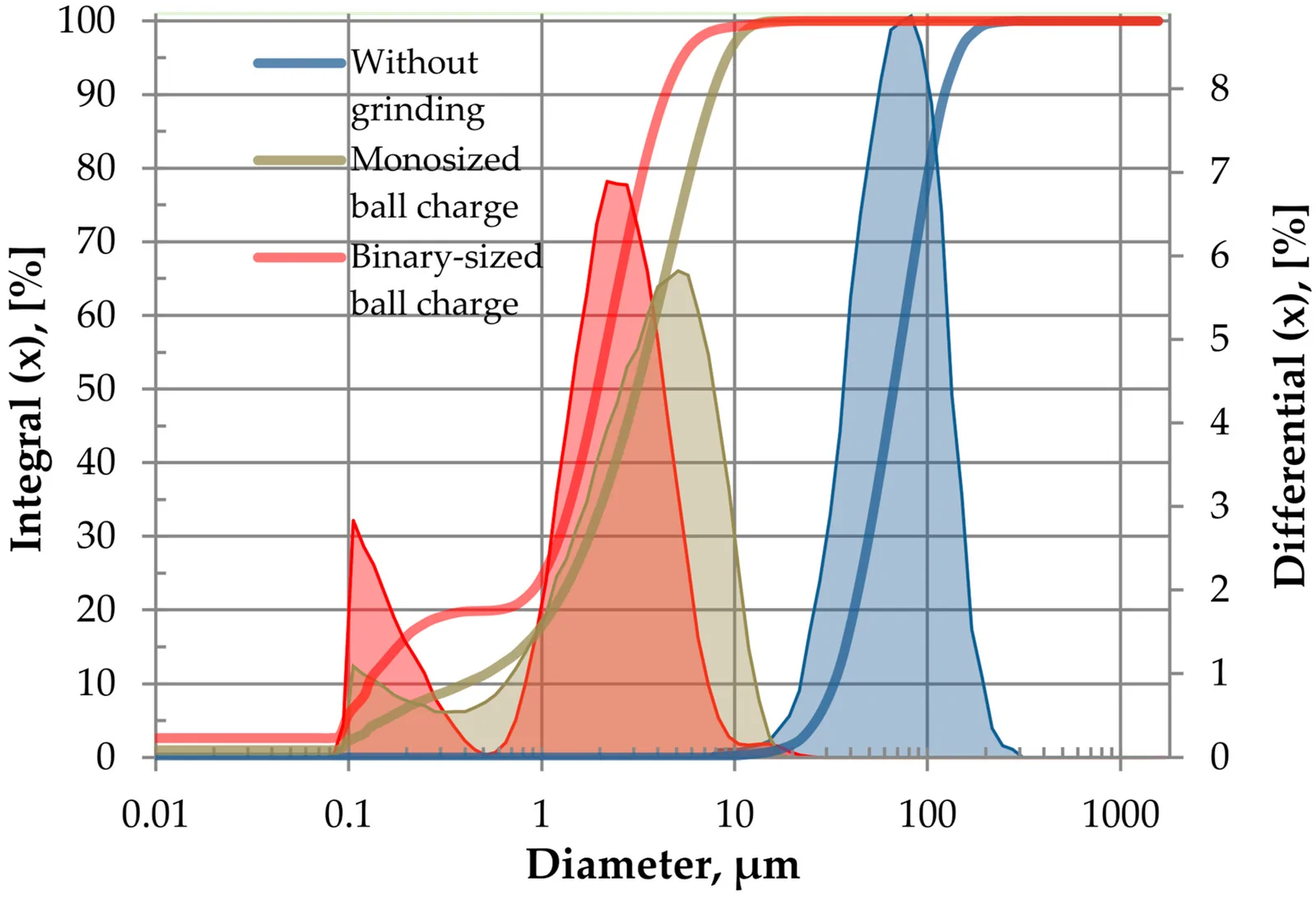
Particle size distribution of the initial B_4_C powders and their milled products in a cryogenic mill with two types of ball feed.

Figure 3 shows similar data for boron carbide. The particle size distribution of the initial B_4_C particles is characterized by a monomodal distribution with a broad range from 10 to 100 μm. The main distribution range is concentrated in particles with a diameter of approximately 50 μm, with particles smaller than 10 μm and larger than 100 μm present in negligible quantities. After cryogenic milling, as with bismuth oxide, the distribution becomes bimodal. The dense, two-fraction ball feed again shifts the primary mode toward smaller sizes—down to 3.9 μm. When using balls of the same diameter, the fine fraction is significantly less pronounced, and the average particle size remains significantly higher—6.5 μm. Furthermore, the use of dense ball loading ensures a narrower particle size distribution and increases the number of submicron grains compared to monosized ball charging. Therefore, binary-sized ball charging is preferred for achieving maximum dispersion of boron carbide.

The use of dense ball charge in pulsed impact compression mode for grinding bismuth oxide and boron carbide is in good agreement with existing data on the mechanical activation of mineral materials [13,14,15,16,17,18]. However, the use of a similar approach for cryogenic milling of polymers has been virtually unknown in the available literature. Therefore, this paper presents a comparative study of the effects of a dense, two-fraction grinding media charge and a monosized ball charge on the grinding efficiency of low-density polyethylene (LDPE) under cryogenic conditions.

**Figure 4:**
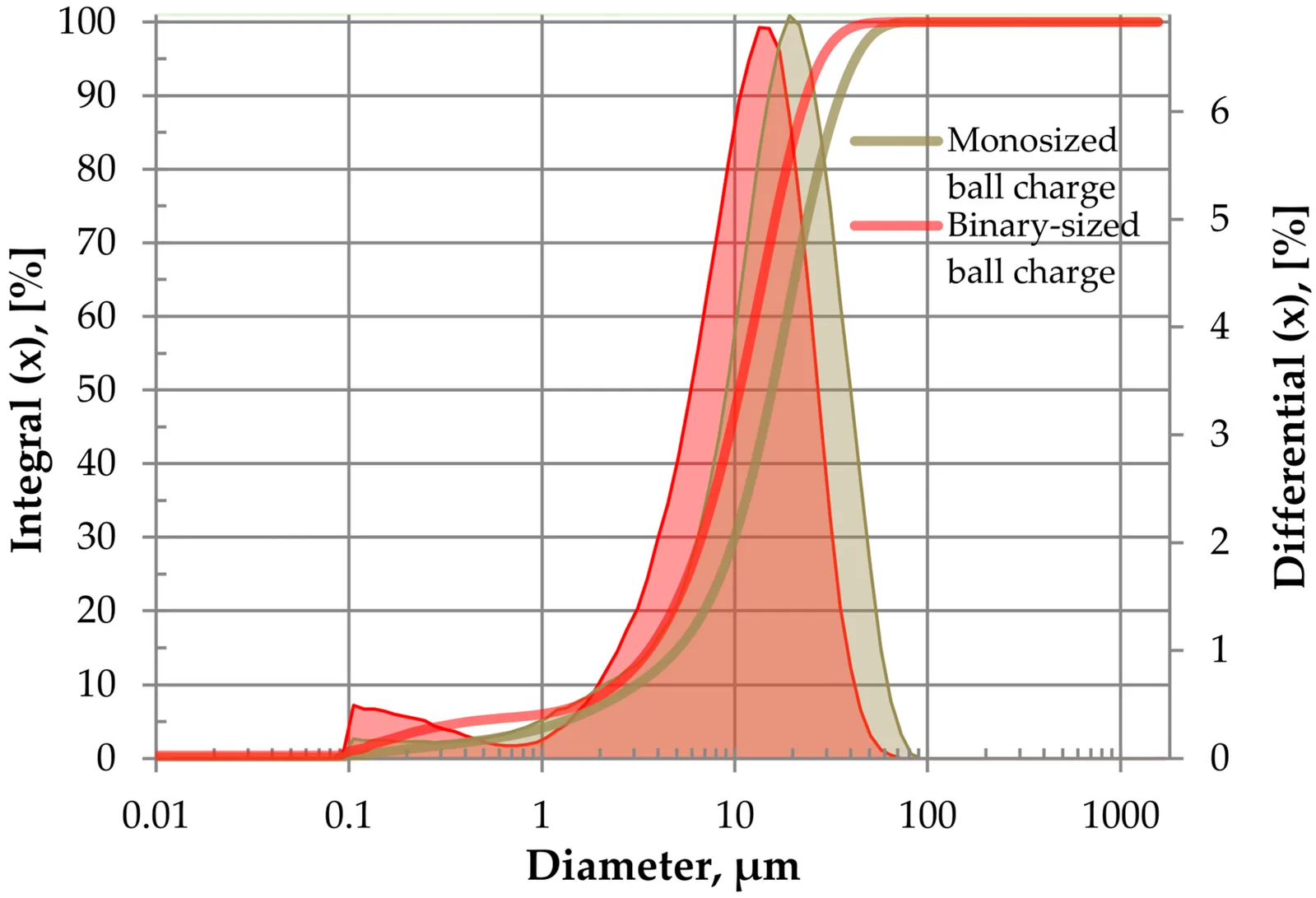
Granulometric composition of LDPE powders after grinding in a cryogenic mill with two types of ball loading.

Figure 4 compares the LDPE particle size distribution curves for samples milled in a cryogenic mill in two modes. Cryogenic treatment using a dense ball charge resulted in a significant reduction in LDPE particle size: the modal diameter was 13.4 μm, and the average volume diameter was 12.1 μm. The distribution span (d90–d10)/d50, which characterizes the degree of uniformity of the particle size distribution, was 1.99, indicating the absence of coarse outliers and a fairly narrow size range. For comparison, when milling in the monosized ball charge mode in a chamber, these parameters were somewhat worse: the modal diameter was 19.1 μm, the average volume diameter was 17.8 μm, and the span was 2.05. Thus, a dense two-fraction loading of grinding media, ensuring the principle of pulsed impact compression, not only intensifies the grinding process, but also ensures a more controlled granulometric composition of both mineral and polymer powders. The results obtained in the present study on the comminution of LDPE particles in the monofractional grinding mode are consistent with other published works [11,25,26].

**Figure 5:**
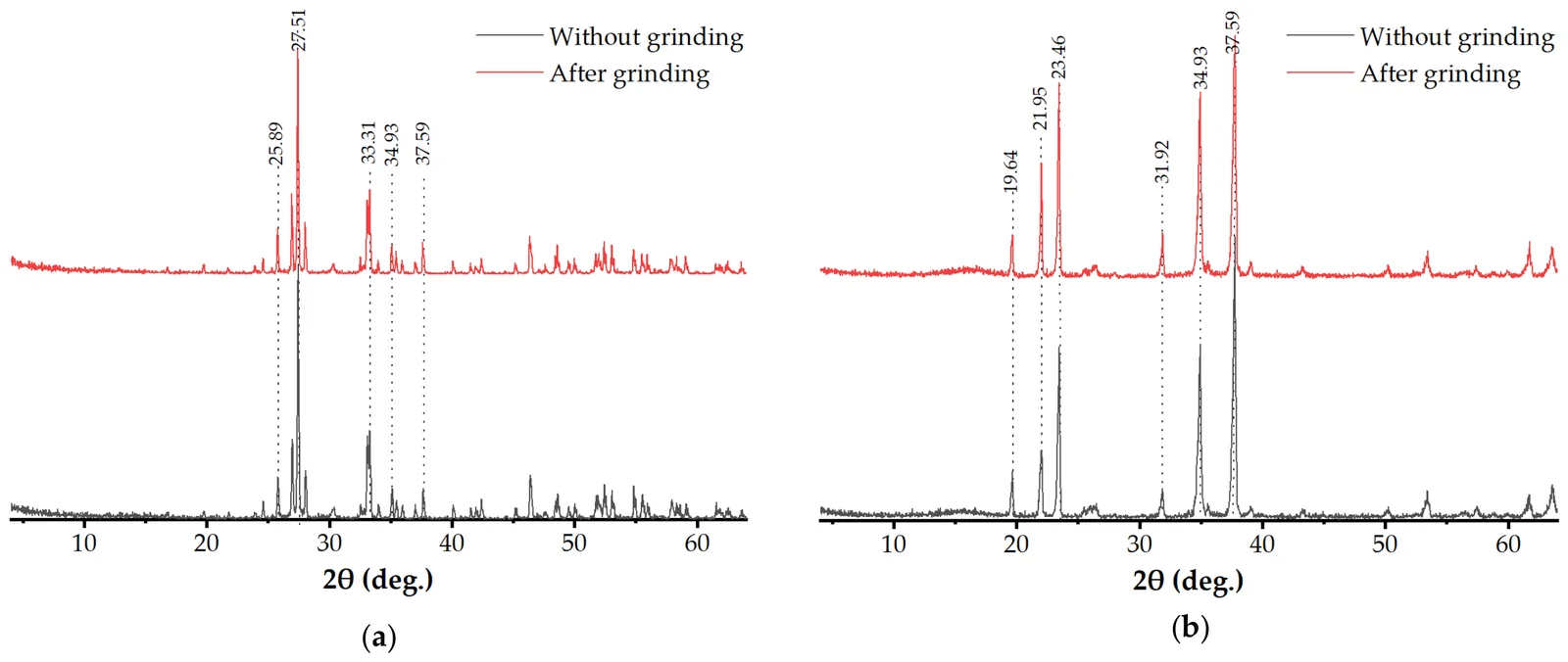
X-ray diffraction patterns of inorganic components before and after cryogenic milling: (**a**) Bi_2_O_3_, (**b**) B_4_C.

After cryogenic milling, the material was compared with the original samples using X-ray diffraction (XRD). The purpose of the analysis was to assess the stability of the crystalline structure of the materials under mechanical stress at extremely low temperatures (below −180 °C) and to identify possible changes that could affect the structure and properties of the resulting composite. Figure 5 shows the X-ray diffraction patterns for the inorganic components.

The bismuth oxide diffraction patterns (Figure 5a) are dominated by an intense reflection at 27.51°, characteristic of Bi_2_O_3_, along with a set of secondary reflections at 25.89°, 33.31°, 34.93°, 37.59°, and other 2θ angles. After cryogenic milling, the intensities and positions of the reflections remain unchanged, indicating the absence of phase transitions or crystallochemical changes in the structure. Thus, mechanical action under cryogenic conditions does not disrupt the Bi_2_O_3_ crystal lattice, and its dispersion can be accomplished without the risk of structural degradation.

A similar pattern is observed for boron carbide. As can be seen from the diffraction pattern (Figure 5b), cryogenic milling had no significant effect on the phase composition or crystal structure. The main reflections, located in the 2θ angle range of 19.64°, 21.95°, 23.46°, 31.92°, 34.93°, and 37.59°, remain identical both before and after milling (Table 1). There are no changes in reflection intensity, or they are within the error limits, indicating preservation of the original crystalline phase. This demonstrates the high resistance of boron carbide to mechanical stress, even at extremely low temperatures.

**Figure 6:**
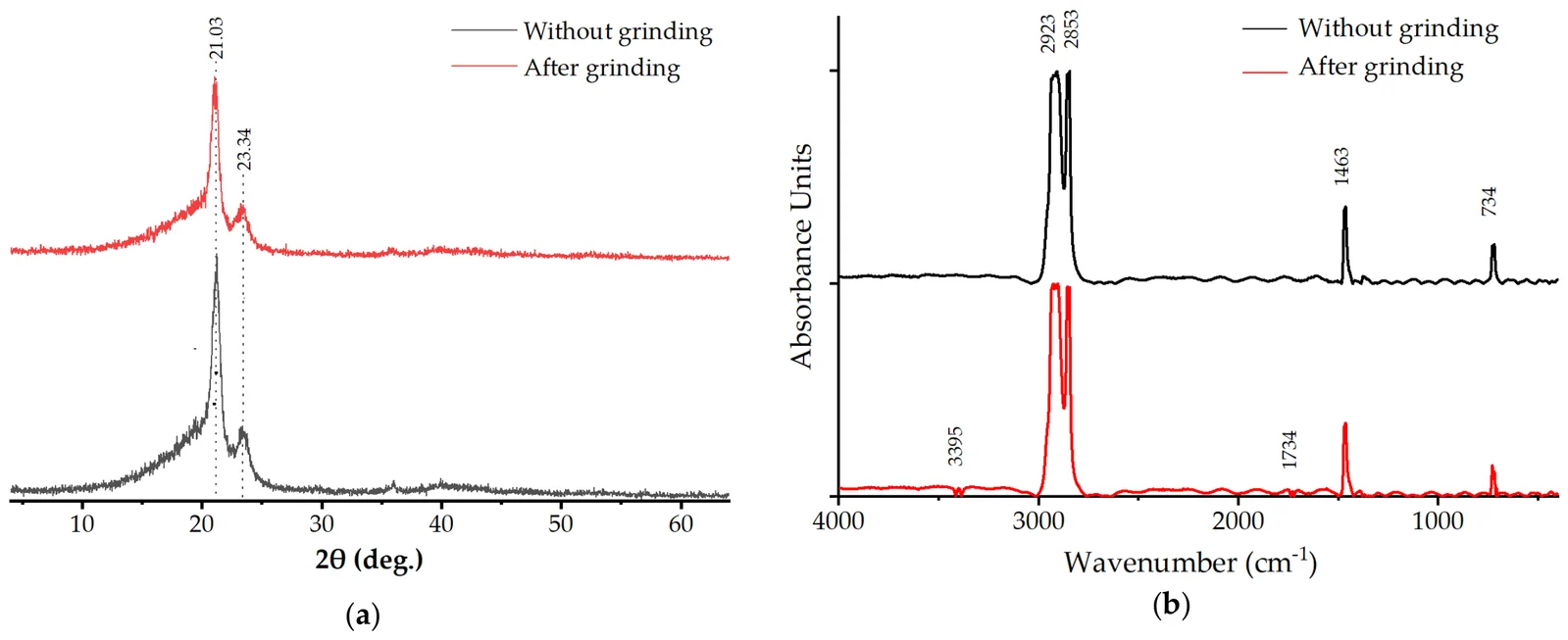
LDPE spectra before and after cryogenic milling: (**a**) XRD, (**b**) IR.

The behavior of LDPE is of particular interest. Unlike inorganic components, it has a partially amorphous structure with a distinct region of crystallinity. X-ray diffraction patterns before and after cryogenic milling (Figure 6a) show changes in the shape and intensity of characteristic broad peaks in the 2θ angle range of 21.03° and 23.34°. After milling, the intensity of the primary signal decreases slightly, with slight broadening and a decrease in its symmetry observed, which may indicate partial destruction of crystallites and growth of the amorphous component. Such changes are typical of polymers subjected to intense mechanical processing and are associated with the destruction of crystalline domains and the ordering of macromolecular chains. Since X-ray phase analysis of LDPE revealed changes in the intensity and shape of diffraction reflections, an additional study using FTIR spectroscopy was conducted to confirm changes in the material’s structure (Figure 6b). For a complete picture, samples before and after processing at temperatures below −180 °C were included in the study.

The FTIR spectrum of LDPE (Figure 6b) after cryogenic milling shows significant changes compared to the original sample. One of the most revealing features is the appearance of an absorption band at 3395 cm^−1^, which corresponds to the stretching vibrations of hydroxyl groups (O–H). This band may be due to both the formation of hydroxyl fragments due to oxidative processes occurring during the cleavage of macromolecular chains and the adsorption of moisture on newly formed active sites in the structure. This band is absent from the original LDPE sample, suggesting that its appearance is a direct consequence of mechanical activation. Furthermore, a new band appears in the spectrum at 1734 cm^−1^, corresponding to carbonyl groups (C=O). This indicates partial oxidation of the polyethylene, leading to the formation of ketones, aldehydes, and other oxygen-containing compounds. These groups are not characteristic of the structure of the original LDPE, further confirming the occurrence of chemical changes due to mechanical disruption of the macromolecules during milling. Additional changes also affect the intensity and shape of existing bands. For example, an increase in the intensity of the band at 1463 cm^−1^, related to deformation vibrations of –CH_2_– groups, is observed, which may be due to disruption of order and an increase in the amorphous component. The band at 734 cm^−1^, associated with the crystallinity of the polyethylene matrix, exhibits a decrease in intensity and partial broadening, indicating the disruption of crystallites and a decrease in the degree of crystallinity. Similar patterns were observed in the study by Huang [27]. Furthermore, Huang noted a non-monotonic change in the degree of crystallinity of HDPE, characterized by an initial decrease followed by an increase and the formation of a monoclinic phase after several milling cycles. In our experiments, the stage of decreasing crystallinity was observed after 30 min of cryogenic milling, which corresponds to the initial phase of mechanochemical transformations described by Huang.

The mechanism of mechanical activation of polyethylene during cryogenic co-milling with fillers is that, at low temperatures, the material becomes brittle and easily fragments into small particles. Mechanical impacts and shearing are accompanied by localized destruction of LDPE macromolecular chains, leading to the formation of radical centers (–CH_2_·) and unsaturated fragments. Concurrently, exposure to oxygen leads to the formation of polar oxygen-containing groups, including hydroxyl (O–H, ~3395 cm^−1^) and carbonyl (C=O, ~1734 cm^−1^), which have been detected spectroscopically. The formation of polar groups potentially facilitates the interaction of polymer molecules with the surface of inorganic fillers. When combined with inorganic substances, the Bi_2_O_3_ and B_4_C particles themselves play an additional role. Due to their high hardness and density, they act as abrasives and micro-grinding media, intensifying localized deformation and polymer degradation. As a result, active sites capable of physicochemical bonding with fillers can form on the surface of the polyethylene matrix, which can contribute to improved interfacial interactions, uniform particle distribution, and stabilization of the composite structure.

**Figure 7:**
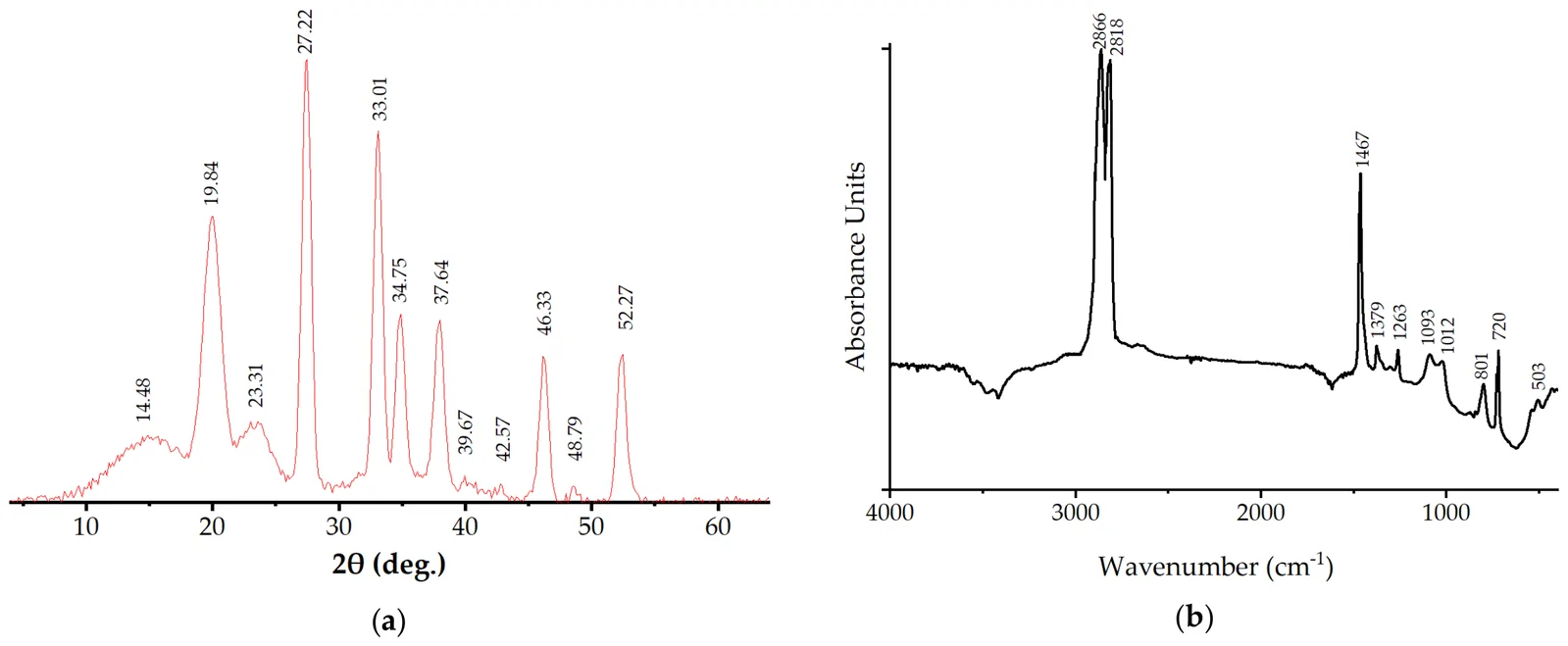
Spectra of composite material: (**a**) XRD, (**b**) IR.

Of particular interest are the structural changes occurring in the material during the formation of a composite product by hot pressing (140–160 °C) from a mixture of components previously subjected to joint cryogenic milling. The X-ray spectrum of the composite (Figure 7a) shows characteristic narrow diffraction reflections confirming the presence of a crystalline structure. Signals at angles of 2θ = 19.84°, 23.31° correspond to polyethylene and indicate the presence of an ordered orthorhombic structure. At the same time, reflections at 27.22°, 33.01°, 34.75°, 37.64°, 39.67°, 42.57°, 46.33°, 48.79°, 52.27° are characteristic of the crystalline structures of bismuth oxide and boron carbide. The presence of such pronounced and multicomponent diffraction peaks indicates a high degree of crystallinity of all components of the composite.

The FTIR spectrum of the composite (Figure 7b) demonstrates both the characteristic bands of polyethylene and the absorption bands due to the presence of fillers—bismuth oxide (Bi_2_O_3_) and boron carbide (B_4_C). The region of 3000–2800 cm^−1^ (C–H stretching vibrations in polyethylene): 2866, 2818 cm^−1^—corresponds to C–H stretching vibrations in the methylene (-CH_2_-) groups of polyethylene. These absorption bands are characteristic of aliphatic hydrocarbons and indicate the preservation of the polyethylene structure in the composite. The region of 1500–1400 cm^−1^ (CH_2_ group vibrations in polyethylene): 1467 cm^−1^—deformation vibrations of CH_2_ groups, characteristic of polyethylene. 1379 cm^−1^ is an additional absorption band, which may be associated with the introduction of fillers into the polyethylene matrix. The region of 1300–1000 cm^−1^ (characteristic vibrations of bonds in composites): 1263 cm^−1^, 1092 cm^−1^, 1017 cm^−1^—these absorption bands can be associated with deformation vibrations of carbon-oxygen (C-O) and carbon-carbon (C-C) bonds, which can arise during the interaction of polyethylene with bismuth oxide. Bismuth oxide can change the structure of molecular bonds, causing the appearance of new absorption bands in this region. The region of 800–400 cm^−1^ (the influence of Bi_2_O_3_ and B_4_C fillers) 801 cm^−1^, 720 cm^−1^—characteristic absorption bands for polyethylene associated with its crystallinity. 503 cm^−1^, 414 cm^−1^—these bands are most likely associated with vibrations of bismuth oxide (Bi-O) and boron carbide (B-C). Bismuth oxide (Bi_2_O_3_) often exhibits characteristic bands in the low-frequency region (400–600 cm^−1^), confirming its presence in the composite. Boron carbide (B_2_C) can manifest itself in the low-frequency region (400–500 cm^−1^) due to vibrations of B–C and Bi–O bonds.

It is also worth noting that during thermal pressing, the previously recorded spectral changes caused by mechanical activation become less pronounced. In particular, a significant decrease in intensity or complete disappearance of absorption bands corresponding to hydroxyl (in the region of 3395 cm^−1^) and carbonyl (approximately 1734 cm^−1^) groups is observed. These phenomena may be associated with thermal relaxation of the polymer structure, the removal of unstable oxidized fragments formed as a result of previous exposure, and the desorption of physically adsorbed moisture. Furthermore, the absorption band at 734 cm^−1^, characteristic of the crystalline phase of LDPE, regains its shape and intensity after heat treatment, indicating partial recrystallization of the polymer matrix. This confirms that some of the structural changes observed after mechanical activation are temporary and reversible and can be ameliorated during thermal stabilization of the material.

**Figure 8:**
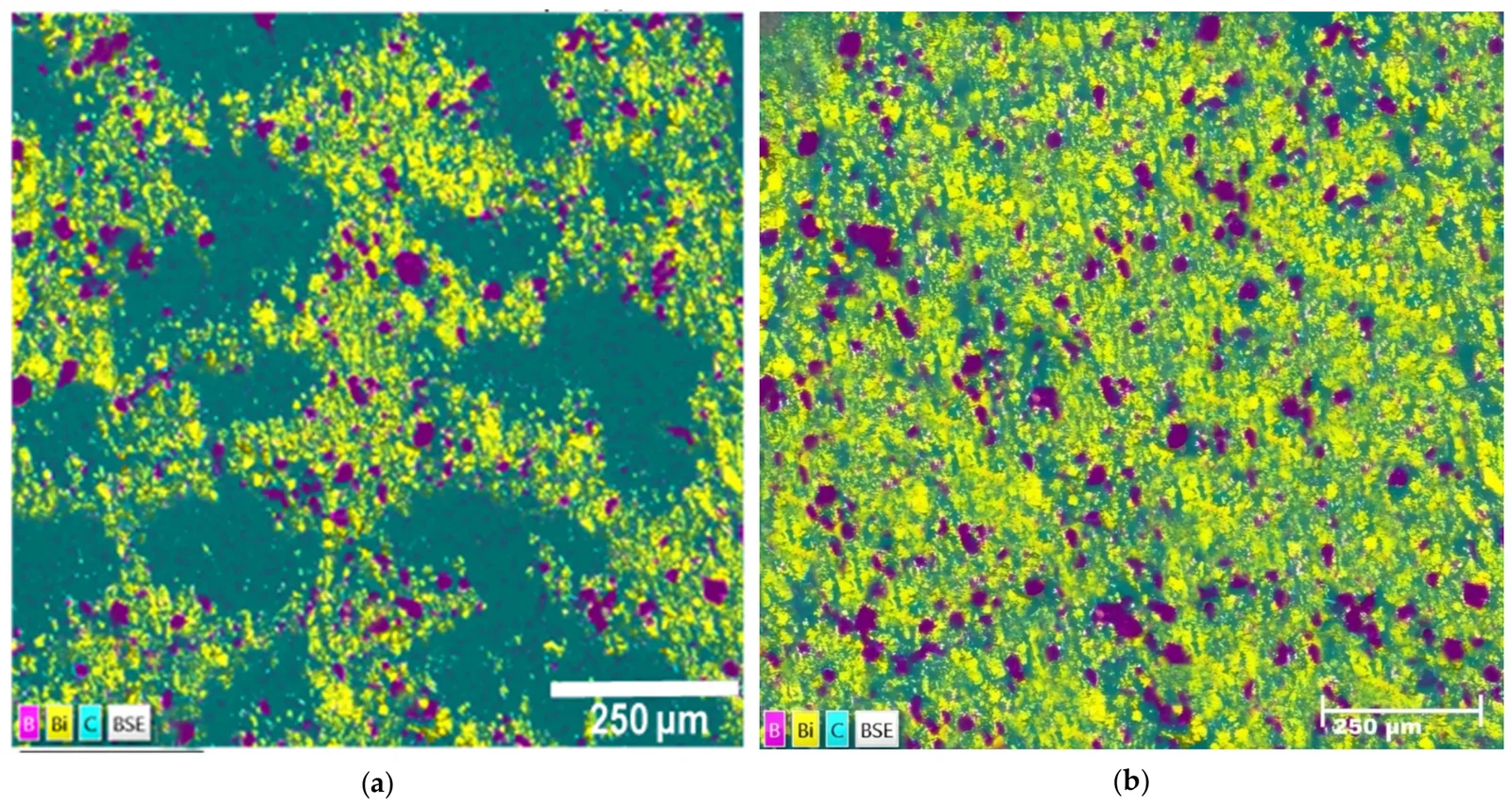
Multilayer EDS map of composite materials from powders prepared by cryogenic milling in the following mode: (**a**) Monosized ball charge, (**b**) Binary-sized ball charge.

To assess the distribution of the inorganic filler in the composite matrix, energy-dispersive spectral analysis (EDS) of the samples was performed (Figure 8). The results show that cryogenic milling using a dense two-fraction ball charge provides a significantly more uniform and homogeneous distribution of filler particles compared to a monosized ball charge. When spheres of a single diameter are used, regions of the polymer phase virtually devoid of inorganic inclusions are clearly visible in the micrographs, whereas such areas are practically absent in the case of dense packing. However, both processing methods are characterized by the presence of both isolated particles and their agglomerates (aggregates), which is apparently due to the high surface energy of fine powders. However, in samples obtained from material prepared using the two-fraction loading technique, the proportion of regions of element atom localization with an area exceeding 500 μm^2^ is lower (Table 2) than in samples obtained with single-fraction loading. Based on the data obtained, it can be concluded that cryogenic milling using a dense, two-fraction ball mill not only reduces the filler particle size but also significantly improves their dispersion in the polymer matrix, which is particularly important for producing materials with reproducible and predictable properties.

Sound velocity measurements (Table 3), performed on five samples of each composition, demonstrated high reproducibility: the velocity values were virtually identical in all cases. This indicates the absence of macrodefects (pores, cracks, delaminations) in the internal structure of the resulting composite materials. At the same time, when comparing composites prepared in binary-sized ball charging mode with samples obtained with single-ball loading, a statistically significant increase in sound velocity was recorded in the former case. This effect is explained by a higher degree of structural homogenization. The dense two-fraction packing of the grinding media ensures a more uniform distribution of filler particles within the polymer matrix and, as a result, reduces the number of localized regions depleted of the inorganic phase. An increase in acoustic wave velocity is a sensitive indicator of improved elastic properties of the material and correlates with the microscopy results presented above on the improvement in the structural homogeneity of the material.

This study was an integral part of a series of works and served as a foundation for subsequent research presented in publications [3,25]. The developed composite material is intended for use in radiation shielding. The combination of Bi_2_O_3_ (gamma-ray shielding) and B_4_C (thermal neutron absorption) within an LDPE matrix provides multifunctional protection. The achieved uniformity of filler distribution directly influences the reproducibility and reliability of the radiation-shielding characteristics. The absence of filler-depleted zones ensures uniform attenuation of ionizing radiation throughout the entire volume of the product.

Mechanical activation has a significant impact on the structure and properties of the composite during its formation. Activation of polyethylene leads to the formation of high-energy sites on the surface of macromolecules, which improves interfacial interactions between the polymer and inorganic phases of the composite. This is especially important in systems containing polar components such as bismuth oxide and boron carbide. Mechanical activation facilitates the adhesion of filler particles to the polymer matrix and reduces the size and number of agglomerates. This directly improves structural homogeneity. As a result, a denser and more stable interfacial boundary is formed, ensuring effective redistribution of mechanical stress.

Under hot pressing conditions, the pre-activated structure demonstrates better component compatibility and higher stability during heating, preventing defects such as microcracks and delamination. Thus, mechanical activation of LDPE before its use in composite materials with bismuth oxide and boron carbide is not only effective but also technologically feasible. Although individual signs of activation are partially eliminated during thermal molding of the product, the compatibility of components achieved at the grinding stage will ensure improved performance characteristics of the composite material, including its radiation and mechanical resistance, structural homogeneity, and increased adhesion of fillers.

## 4. Conclusions

Dense two-fraction loading of balls during cryogenic grinding of polyethylene with inorganic fillers is a rational technological method that ensures a high degree of homogenization of the mixture and, as a consequence, the formation of composite materials with a controlled particle size distribution and stable performance properties. This method does not require the use of organic solvents, which makes it an environmentally friendly technological approach.

It was shown for the first time that the use of binary (two-fraction) ball loading in a cryogenic mill provides more efficient dispersion of low-density polyethylene and inorganic fillers (Bi_2_O_3_ and B_4_C) compared to monofraction loading. The proportion of particles smaller than 5 μm increases by 1.25 times for Bi_2_O_3_, and the modal diameter of LDPE decreases from 19.1 to 13.4 μm. It has been established that cryogenic grinding leads to mechanochemical destruction of polyethylene, accompanied by the formation of hydroxyl and carbonyl groups (IR spectroscopy data). However, subsequent hot pressing neutralizes these changes, which indicates the reversible nature of the oxidative processes. The EDS mapping method confirmed that the proposed mixing mode makes it possible to achieve a more uniform distribution of fillers in the matrix, reducing the number of zones depleted in the inorganic phase. An increase in ultrasound speed by ~3.5% (from 2038 to 2110 m/s) indicates an increase in the structural homogeneity and elastic properties of the composite.

The proposed method is scalable and can be integrated into existing polymer composite production lines, as it does not require organic solvents, high temperatures, or additional dispersing agents. Key advantages of this approach over existing alternatives include the combination of grinding and mixing processes into a single technological cycle, which reduces production costs and eliminates the need for separate component preparation. Furthermore, this approach enables the production of submicron-sized filler particles without additional wet milling stages. However, it should be noted that the size and ratio of grinding media must be carefully selected for other composite systems, necessitating additional calculations and preliminary experiments.

## Figures and Tables

**Table 1:** X-ray parameters of materials.

Materials	Without Grinding				After Grinding			
	Angle 2θ, deg.	d, Å	I/I_max_	FWHM	Angle 2θ, deg.	d, Å	I/I_max_	FWHM
Bi_2_O_3_	25.80	3.450	0.165	0.12	25.76	3.456	0.205	0.12
	27.44	3.248	1.000	0.13	27.40	3.253	1.000	0.11
	33.28	2.706	0.367	0.23	33.28	2.707	0.392	0.24
	46.39	1.956	0.211	0.14	46.33	1.958	0.207	0.13
B_4_C	23.44	3.792	0.645	0.19	23.42	3.795	0.733	0.17
	34.92	2.567	0.694	0.21	34.94	2.566	0.741	0.19
	37.74	2.382	1.000	0.20	37.74	2.382	1.000	0.20
LDPE	21.20	4.187	1.000	0.75	21.06	4.218	1.000	0.93
	23.38	3.801	0.280	1.12	23.24	3.827	0.279	1.31

**Table 2:** Proportion of regions of element atom localization with an area exceeding 500 μm^2^.

Component Pre-Preparation Mode	B	C (Polymer Phase)	Bi
Monosized ball charge	0.46	0.36	0.85
Binary-sized ball	0.24	0.31	0.74

**Table 3:** The passage of the speed of sound in the materials under study [25].

Component Pre-Preparation Mode	Speed of Sound in Material (m/s)					Modulus of Elasticity: E, MPa
	Sample Number					
	1	2	3	4	5	
Monosized ball charge	2032	2038	2045	2039	2035	2.36
Binary-sized ball charge	2102	2094	2114	2112	2109	3.34

## Data Availability

The original contributions presented in this study are included in the article. Further inquiries can be directed to the corresponding author.
